# Evaluation of the Fracture Resistance of Different Designs of All-Resin Post and Core Systems: An In Vitro Study

**DOI:** 10.7759/cureus.54137

**Published:** 2024-02-13

**Authors:** Mosa Altassan, Osamah Alsulimani, Bashayer M Alzahrani, Abdulmajeed Alghanemi, Abdullah Abukhudhayr, Shooq Alharbi, Nabeel Munshi

**Affiliations:** 1 Department of Oral and Maxillofacial Prosthodontics, Faculty of Dentistry, King Abdulaziz University, Jeddah, SAU; 2 Department of Oral Diagnostic Sciences, Faculty of Dentistry, King Abdulaziz University, Jeddah, SAU; 3 Department of General Dentistry, Faculty of Dentistry, King Abdulaziz University, Jeddah, SAU

**Keywords:** fracture resistance, post length, glass fiber post, all-resin post, post and core

## Abstract

Introduction

There is a growing demand for post and core systems that offer both ease of use and efficiency. Recently introduced dual-cure build-up and post cement materials exhibit properties similar to dentin. The objective of this laboratory experiment is to compare the fracture resistance among three distinct post and core systems and identify the locations of failures within each group.

Material and methods

This in vitro experimental study involved 30 epoxy resin-based blocks (Endo Training Bloc J, Dentsply Sirona, Ballaigues, Switzerland) divided into three groups: The first group was a post space preparation and restoration with a fiber post (RelyX™ Fiber Post, 3M ESPE, Saint Paul, Minnesota, United States) 1.6 mm in diameter and 10 mm in length (Group A) where core build-up and cementation were performed using a dual-cure build-up and cement for endodontic post resin material (Core X Flow, Dentsply DeTrey, Konstanz, Germany). The second group was a post space preparation and restoration using a dual-cure build-up and cement for endodontic post resin material, 10 mm in length filled with resin but without fiber post placement (Group B). The third group was where post space and core were filled and restored with a dual-cure build-up and cement for endodontic post resin material, 5 mm in length and without fiber post placement (Group C). Subsequently, samples were mounted and tested using a universal testing machine (Instron, Canton, Massachusetts, United States), and the fracture site was located.

Results

Significant differences were identified among the three groups, indicating the impact of both post length and type on fracture resistance (p-value <0.05). Group B exhibited the highest mean compressive strength resistance and maximum load at 899.3330 (N), followed by Group C at 848.9690 (N) and Group A at 751.9620 (N). The predominant failures in the samples were core fractures or debonding of the core material.

Conclusion

All-resin posts demonstrated high fracture resistance, unlike fiber posts which displayed inferior fracture resistance.

## Introduction

The most critical factor for the long-term success of compromised, endodontically treated teeth (ETT) is the preservation of the remaining sound tooth structure [1]. If the remaining tooth structure is compromised, it will be unable to receive a permanent prosthesis. Thus, a post becomes necessary to retain the core, regardless of post length and type [2,3]. Currently, numerous post and core systems are available, differing in post type, design, surface texture, fit, and material [4].

For a considerable time, cast post and core have conventionally been the primary treatment choice for compromised teeth following endodontic procedures. However, recent advancements in the biomechanics of fiber-glass posts, attributed to their aesthetic appeal and reduced chairside working time, have led to a shift in preference. Moreover, their similarity to dentin in terms of modulus of elasticity aids in decreasing the likelihood of root fractures due to stresses, thereby improving the prognosis of the prosthesis. Consequently, they are now considered a favorable option in contemporary practice [2].

The history of fiber posts began in the early months of 1989, when the inventors claimed to introduce their original carbon fiber post-composite post, utilizing it clinically in France [5]. Fiber post exhibits a modulus of elasticity that is close to dentin which relatively promotes even distribution of stresses, consequently reducing the risk of root fracture [6]. Moreover, some researchers argue that the presence of posts could potentially disrupt the mechanical resistance of a tooth that has undergone root canal treatment, thereby increasing the risk of mutilation to the remaining tooth structure [7]. Currently, there is no unanimous agreement on the optimal material or technique for restoring these ETT, which are particularly susceptible to fractures [8].

Newly introduced dual-cure build-up and post cement materials exhibit properties similar to dentin, such as strength, flexibility, and insulating qualities. Responding to the demand for clinical convenience in tooth structure restoration, these materials envelop posts seamlessly, eliminating gaps or voids [9]. Resin cements share a composition resembling that of composite resin but with lower filler content. The correlation between filler content and physical/mechanical properties is noteworthy. Higher filler content enhances hardness, stiffness, durability, and strength while minimizing shrinkage during polymerization. However, the lower viscosity of resin cements compared to composite resins heightens shrinkage stress, increasing the risk of debonding and microleakage [10].

In the realm of post and core systems, there is a growing demand for post and core systems that provide ease and efficiency in the restoration of missing tooth structures. This need has led to the development of diverse "one-visit" alternatives for post and core restorations [11,12]. In this study, two designs of a new post system will be introduced and tested for the first time in an in vitro setting. The resin material utilized for build-up and cementation will be employed as a post system, a usage that, despite being observed in some clinical practices, lacks supporting evidence.

Thus, the authors have proposed two systems with variations in length, specifically 5 mm and 10 mm, both utilizing Core X® Flow Dual Cure Core Build-Up Material and Cement for Endodontic Posts with the utilization of epoxy resin blocks to explore the feasibility of using core build-up material as a post in comparison to prefabricated glass-fiber post as a control. As per the manufacturer, this material is composed of urethane dimethacrylate, di- and tri-functional methacrylates, barium boron fluoroaluminosilicate glass, camphorquinone (CQ) photoinitiator, photoaccelerators, silicon dioxide, and benzoyl peroxide.

As a result, it will provide clinicians and researchers with an evidence-based foundation for future studies on natural teeth. Additionally, this laboratory experiment aims to compare the fracture resistance of three different post and core systems while identifying the types of failures in different groups. The null hypothesis suggests that no significant differences would be observed among the three groups, in terms of both mechanical testing and the type of fracture.

## Materials and methods

This in vitro study was conducted at King Abdulaziz University in Jeddah, Saudi Arabia. Ethical approval was obtained from the Research Ethical Committee of the Faculty of Dentistry, King Abdulaziz University (approval number: 4493718). A total of 30 epoxy resin blocks (Endo Training Bloc J, Dentsply Sirona, Ballaigues, Switzerland) were selected to conduct this study. Since it is the first time to use all-resin posts, the epoxy resin blocks seemed to be more convenient to maintain the standardization of the experiment and eliminate any anatomical variations, lengths, and geometrical differences that are seen in natural teeth.

The resin blocks were instrumented with hand files up to size #20 K-file as glide path followed by rotary files up to F3 (ProTaper Gold, Dentsply Sirona, Ballaigues, Switzerland). After irrigation with normal saline to remove resin debris, obturation was performed using F3 matching gutta-percha points using a single-cone technique (Gutta Percha Points, Meta Biomed Co., Ltd., Chungcheongbuk-do, Korea) and sealed with a resin-based root canal sealer (Adseal™, Meta Biomed Co., Ltd., Chungcheongbuk-do, Korea). All the samples were randomly assigned to three groups of 10 using Random Allocation Software 2.0.

**Figure 1:**
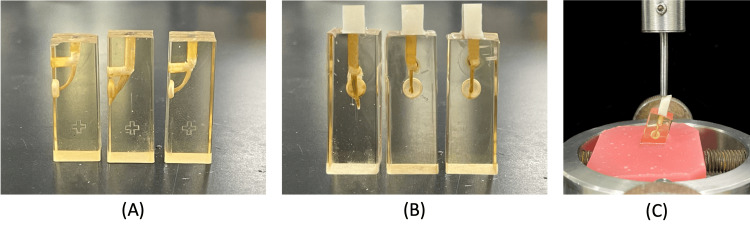
(A): Post space preparation and holes in the middle were made for mechanical retention. (B): Post cementation and core build-up. (C): Samples mounted at 45 degrees and subjected to compressive force

After 24 hours, post space was prepared using Gates Glidden Drills sizes #2 and #3 (Dentsply Sirona, Ballaigues, Switzerland) as shown in (Figure 1(A)). The hole in the middle portion of the epoxy resin blocks was made with diamond burs to enhance retention by mechanical interlock of the core build-up material with the epoxy resin blocks. Furthermore, petroleum jelly was used as a separating medium (Vaseline, New York, New York, United States) to eliminate any bonding effect between post and core restoration and the resin blocks. All groups were named and restored as follows: Group A was a post space preparation and restoration with a fiber post (RelyX™ Fiber Post, 3M ESPE, Saint Paul, Minnesota, United States) 1.6 mm in diameter and 10 mm in length where core build-up and cementation were performed using a dual-cure build-up and cement for endodontic post resin material (Core X Flow, Dentsply DeTrey, Konstanz, Germany). Group B was a post space preparation and restoration using a dual-cure build-up and cement for endodontic post resin material (Core X Flow, Dentsply DeTrey, Konstanz, Germany), 10 mm in length filled with resin but without fiber post placement. Group C was a post space preparation and restoration with a dual-cure build-up and cement for endodontic post resin material (Core X Flow, Dentsply DeTrey, Konstanz, Germany) 5 mm in length without fiber post placement.

The application of the resin material for fiber post cementation and light curing was done according to the material's manufacturer instructions (MiniLED Standard, Acteon, Mérignac, France) for 20 seconds. For Groups B and C, the material was injected directly inside the canal to the outside and cured for 10 seconds. The build-up was done around the extended part and then cured for 20 seconds with the same light-curing device. Cores were finished using a finishing disk and beveled as shown in Figure 1(B) and standardized to a length of 8 mm and a width of 3 mm.

**Figure 2:**
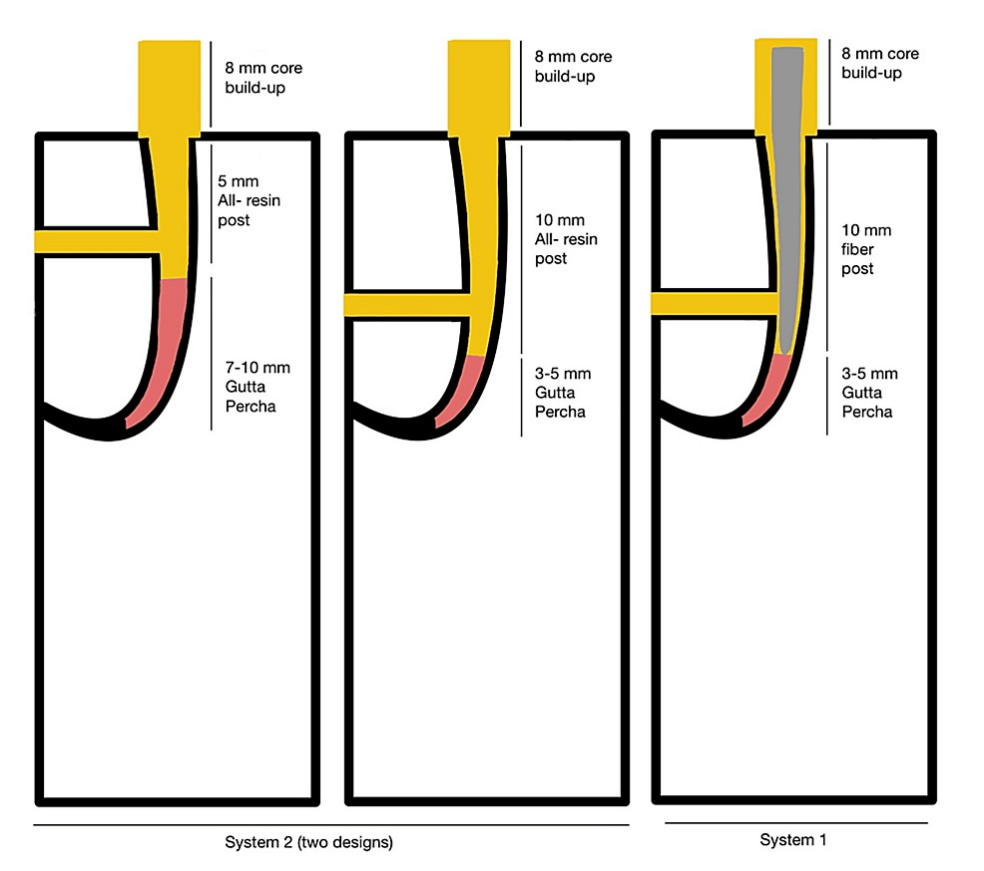
Illustration of the fiber post system design and all-resin system designs

**Figure 3:**
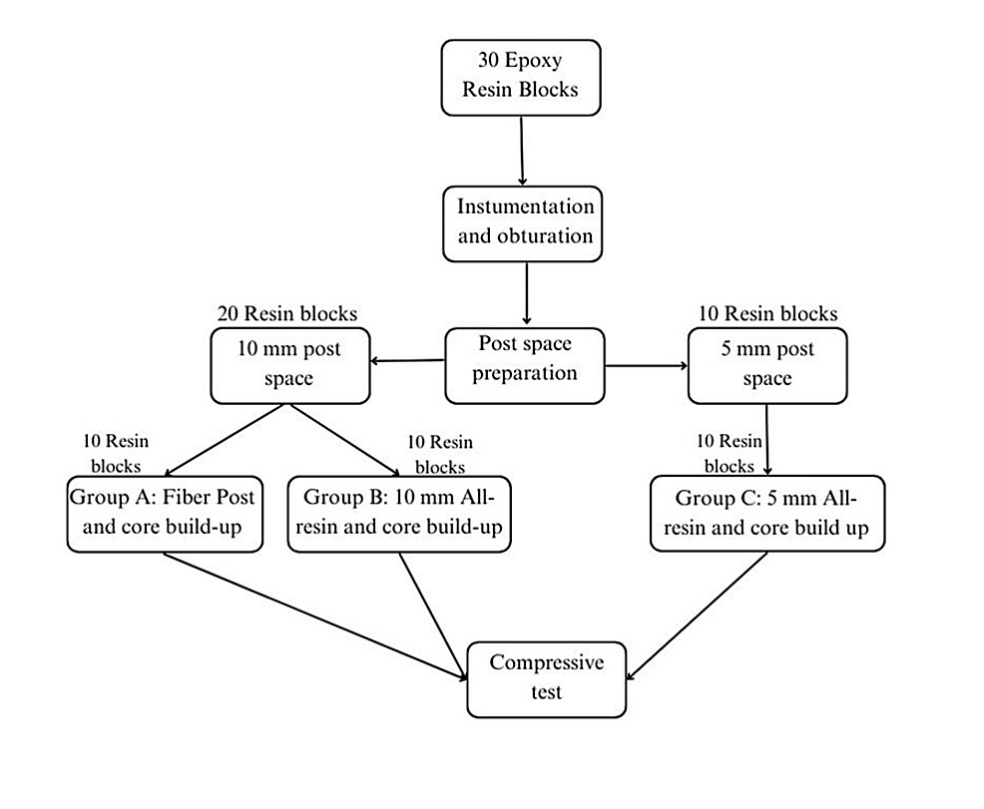
Flowchart summarizing the experiment

The samples were then placed in an acrylic base with a 45-degree angulation to mimic stress-generating forces, such as lateral forces. Finally, the samples underwent compressive strength testing using the universal testing machine (Instron, Canton, Massachusetts, United States) until a fracture occurred, either to the core alone or to the post and core (Figure 1(C)). Due to the elimination of the bonding effect, compressive strength testing was the most suitable for this sample. Both system designs are illustrated in Figure 2. The experiment was summarized in a flowchart shown in Figure 3. 

Statistical analysis

IBM SPSS Statistics for Windows, Version 26.0 (Released 2019; IBM Corp., Armonk, New York, United States) was used for statistical analysis. The results were analyzed using the one-way analysis of variance (ANOVA) followed by Tukey's Honest Significant Difference (HSD) test.

## Results

Table 1 shows the mean and standard deviation of the forces applied (failure point load) of the three groups: Group A (prefabricated fiber post with 10 mm length: control group), Group C (all-resin post with 5 mm length), and Group B (all-resin post with 10 mm length). Statistical analysis conducted using the one-way ANOVA test revealed significant differences between the three groups (p-value <0.05) as shown in Table 2. 

**Table 1:** Mean and standard deviation in N N: newton

	N	Mean	Std. deviation	Std. error	95% confidence interval for mean		Minimum	Maximum
					Lower bound	Upper bound		
Group A	10	751.9620	9.99539	3.16082	744.8117	759.1123	737.43	770.54
Group B	10	899.3330	6.27873	1.98551	894.8415	903.8245	890.54	910.34
Group C	10	848.9690	3.99081	1.26201	846.1141	851.8239	843.13	855.64
Total	30	833.4213	62.59181	11.42765	810.0492	856.7935	737.43	910.34

**Table 2:** The impact of post length and post type on the failure loads using one-way ANOVA ANOVA: analysis of variance

	Sum of squares	df	Mean square	F	Sig.
Between groups	112217.007	2	56108.504	1084.173	.000
Within groups	1397.313	27	51.752		
Total	113614.320	29			

Multiple comparisons were done using Tukey's HSD test (Table 3). After subjecting all 30 samples to the universal testing machine (Instron), experimental results indicated that Group B exhibited the highest mean compressive strength and maximum load (899.3330 N), followed by Group C (848.9690 N) and Group A (751.9620 N), respectively.

**Table 3:** Tukey's HSD test *: the mean difference is significant at the 0.05 level; HSD: Honest Significant Difference

(I) group	(J) group	Mean difference (I-J)	Std. error	Sig.	95% confidence interval	
					Lower bound	Upper bound
A	C	-97.00700^*^	3.21721	.000	-104.9838	-89.0302
	B	-147.37100^*^	3.21721	.000	-155.3478	-139.3942
B	C	50.36400^*^	3.21721	.000	42.3872	58.3408
	A	147.37100^*^	3.21721	.000	139.3942	155.3478
C	B	-50.36400^*^	3.21721	.000	-58.3408	-42.3872
	A	97.00700^*^	3.21721	.000	89.0302	104.9838

Visual observation of fracture locations showed that the most common failures involved core fracture only or detachment of the core material. In the fiber post group, fractures were exclusively in the core part. Conversely, in the all-resin group, core fractures occurred with a depth of 1 mm in the canal. The significance of the results suggests a comparable fracture resistance difference between the fiber post material and Core X Flow material. Moreover, the most prevalent failure in the samples involved core fractures only.

## Discussion

Fractures in ETT primarily result from alterations in tooth structural integrity due to factors such as access preparation, diminished proprioception, and reduced tactile sensitivity. These considerations make the final restoration of ETT a critical aspect of dental care. The growing preference for tooth-colored posts has led to an increased adoption of non-metallic posts. The evolution of dental materials has fundamentally transformed post and core systems, facilitating a shift from mechanically retained restorations to adhesively retained ones. These advancements, coupled with a variety of material options, introduce complexity into clinical decision-making concerning ETT replacement, including the choice of whether or not to use posts and the selection of appropriate materials [6].

**Figure 4:**
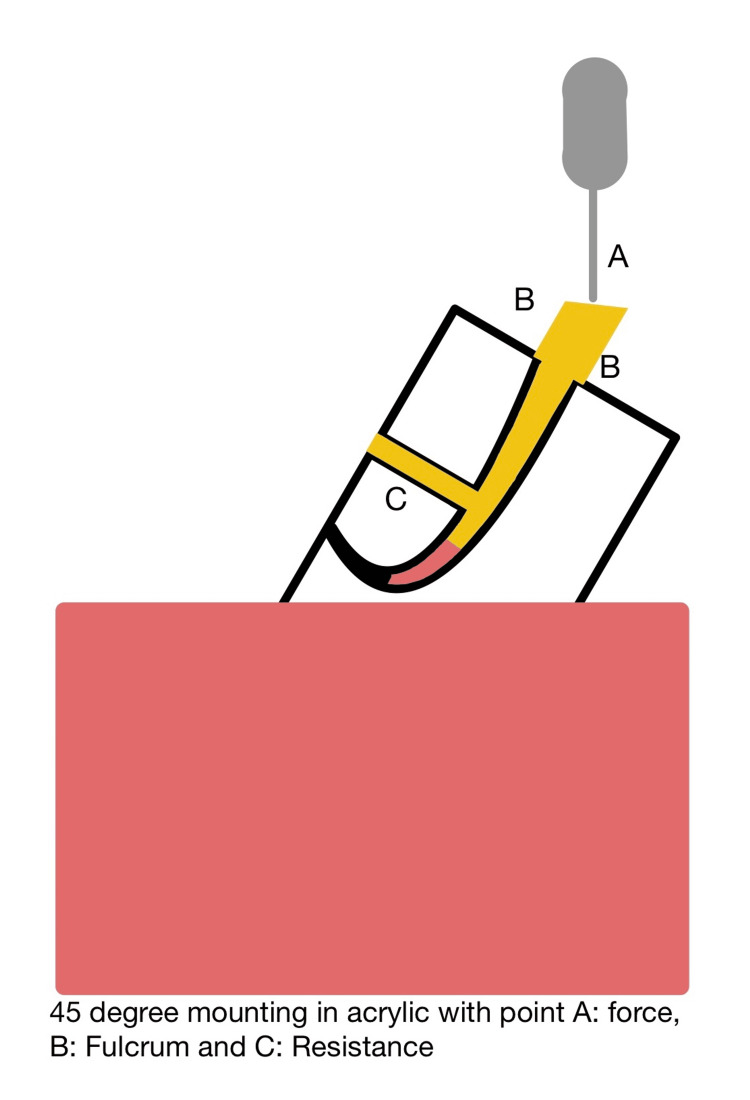
Class I lever arm resembling a three-point bending test

Several factors, including the type and design of the post, the preparation of post space, the choice of luting cement, and the procedures involved in cementation, can significantly impact post retention. According to post space preparation guidelines, the post diameter should be equal to one-third of the root width, with the length related to the root length equal to two-thirds of the root length. Additionally, the post length related to the crown height should be equal to or greater than the crown height. Finally, the length of the remaining gutta-percha should range from 3 mm to 5 mm, with no space left between the post and root canal filling [13]. The post system in this study was designed in accordance with these guidelines, with the post space diameter standardized to 1.6 mm in all samples. Different post lengths will change the stress distribution and will act as Class I lever arm; therefore, it will resemble a three-point bending test with different levels of resistance points (Figure 4).

Despite the anticipated interference of endodontic procedures and materials with the adhesion of resin-based cements, the utilization of adhesive cementation is expected to enhance both post retention and the resistance of roots to fractures [14]. This enhancement is particularly notable when using an all-resin post and core system. Furthermore, there has been a significant shift in the understanding of post and core systems, driven by advancements in dental materials. This transformation has resulted in the widespread adoption of adhesive-dependent restorations over mechanically retained ones [6]. Nevertheless, the preparation of post space continues to be viewed as an invasive procedure, creating a dilemma for some clinicians who must decide whether to restore ETT with or without a post. Conversely, the increasing popularity of core build-up materials is attributed to their straightforward clinical procedures and their ability to conserve tooth structure [15].

Additionally, various resin composite core build-up materials differ in composition, resulting in variations in their chemical and mechanical performance [16,17]. The selection of core build-up material significantly influences the durability of prosthetic restorations. Resin composites have undergone continuous enhancement in their properties through ongoing modifications to their composition since their introduction to the market. Resin composites generally consist of an organic polymer matrix, predominantly inorganic fillers, coupling agents to establish a chemical bond between organic and inorganic components, initiators, inhibitors, accelerators, and additional additives. The innovation of dual-polymerizing resin composites, incorporating both light and self-polymerization initiators, has broadened the applications of this material group. Consequently, adhesive luting of endodontic posts and subsequent core build-up can now be achieved with a single material, reducing treatment times and minimizing potential weaknesses in material interfaces and processing errors [18].

Clinicians actively seek simple, convenient, and durable post types that endure in the patient's oral cavity without adverse effects, and if complications arise, they should be repairable. The fiber post aligns with these criteria effectively. An unconventional approach involves using core build-up material as a post without incorporating a prefabricated glass fiber post. Remarkably, no studies have explored this newly introduced post and core system. If proven effective, it could revolutionize perspectives on post and core restorations. The objective of the study is to compare the fracture resistance of post systems across different lengths, considering the location of fractures. This evaluation aims to ascertain if we can minimize invasiveness in preparing post and core restorations. Additionally, several studies have indicated that the biomechanical performance of cast posts and cores, as well as stainless steel and fiber posts, remains unaffected by the length of the post, contrary to others [19]. 

This in vitro experiment showed that Group B exhibited superior performance compared to Groups A and C. The findings provide evidence to reject the null hypothesis suggesting that there is no statistically significant difference in the fracture resistance among the three groups. In addition, the location of failures was similar among the groups. A statistical analysis of fracture resistance values between the control group (prefabricated glass fiber post) and trial groups (all-resin post and core 5 mm and 10 mm) showed a high statistical significance (p-value <0.05). Therefore, the use of all-resin post and core and the post length had a direct relation to the fracture strength of ETT. In this experiment, the authors avoid the variations of tooth conditions, such as root length, amount of tooth structure left, geometrical design of the tooth, structure integrity, tooth aging, tooth size, and tooth decomposition after extraction. As a result, instead of using natural teeth, all samples can be standardized with the same measurements using epoxy resin-based blocks that were introduced for the first time to be used for post materials' mechanical testing due to their ability to mimic the root canal and help to get clearer and more standardized results. The cores were finished using a finishing disk and beveled at 45 degrees in the core of the samples to avoid pin slippage of the universal testing machine (Instron) when the vertical force was applied. Unlike the other experiment, the sample core was flat. The samples were then placed in an acrylic base with a 45-degree angulation. Additionally, a cold-cured acrylic resin (PalaXtreme®, Kulzer GmbH, Hanau, Germany) base was used to stabilize the samples in the desired angulation, without causing base deformation from the applied force. This 45-degree angulation was chosen to mimic stress-generating forces, such as lateral forces. 

The results of the collected data revealed that the all-resin post, introduced for the first time, can serve as an alternative and be placed inside the canal without the need for a glass fiber post in some clinical cases. Additionally, severely curved canals such as S-shaped, C-shaped, and dilacerated roots, which pose difficulty in post preparation, can be more easily restored with the all-resin post without post space preparation due to the flowability of the material. Unfortunately, this technique has some disadvantages. Firstly, root canal retreatment might be more challenging and could increase the risk of perforation or gauging while removing the all-resin material, owing to the resin shade that makes it hard to locate the canals and makes cleaning and shaping more difficult. Consequently, the procedure may become more challenging and time-consuming. The reliability of this in vitro study underscores the need for further research. This data can be used as a baseline to establish a background for subsequent studies. The need for the use of natural teeth is inevitable and should be confirmed with case reports to support evidence-based clinical practice. This all-resin technique might be revolutionary and mark a significant advancement in restorative and prosthodontics dentistry. It has the potential to improve treatment outcomes on various fronts and further reduce chairside working time.

This in vitro experiment excluded bonding factors such as the use of any bonding system, which is not applicable due to the use of epoxy resin blocks, unlike natural teeth. As a result, the retention of the post and core build-up materials was dependent on mechanical interlock unlike other similar experiments, which might be a limitation. Other limitations include the small sample size and the in vitro nature of the study which was hardly mimicking the oral environment. Nevertheless, the samples were made of epoxy resin, not natural teeth, which doesn't represent the natural teeth condition. Moreover, epoxy resin blocks might not have any impact on the experiment, most probably due to there being no bonding factors, and the results can be reliable. In addition to that, the shape of the post and core might slightly vary between samples. In the post area, the cleaning and shaping step might affect the shape of the post space. For the core part, the used finishing disks might cause some core size variations. The last limitation was mimicking chewing forces precisely, which is noted in lateral glides. It was partially achieved by mounting the resin block at 45 degrees.

## Conclusions

Within the limitations of this in vitro study, it is concluded that the length and type of the post placed affect fracture resistance. All-resin posts demonstrate high fracture resistance, unlike fiber posts which showed lower fracture resistance. Group B (all-resin post with 10 mm length) exhibited the highest mean of fracture resistance and maximum load (899.3330 N), followed by Group C (all-resin post with 5 mm length) (848.9690 N) and Group A (prefabricated fiber post with 10 mm length: control group) (751.9620 N), respectively. These findings provide a basis for initiating further in vitro experiments on natural teeth to ascertain the applicability of the all-resin post system.
